# YOLO-FSEP: An Improved YOLOv8n Algorithm for Sugar Orange Detection in Orchards

**DOI:** 10.3390/s26123848

**Published:** 2026-06-17

**Authors:** Tianfa Deng, Jinchao Sun, Qingjuan Zhao, Faguo Huang

**Affiliations:** Key Laboratory of Advanced Manufacturing and Automation Technology (Guilin University of Technology), Education Department of Guangxi Zhuang Autonomous Region, Guilin 541006, China; 18374695431@163.com (T.D.); 18052399135@163.com (J.S.); 15578379505@163.com (F.H.)

**Keywords:** YOLOv8n, sugar orange detection, SCSA attention mechanism, Focal_SIOU loss function, P6 detection head

## Abstract

**Highlights:**

**What are the main findings?**

- YOLO-FSEP, an improved YOLOv8n-based algorithm integrating SCSA attention, Focal_SIOU loss, SE attention, and a P6 detection head, achieves 71.7% mAP50-95 (3.2% improvement) at 62.6 FPS for sugar orange detection in complex orchard environments.
- A 200-frame continuous test on a laptop with a RealSense D435i camera gives 49.90 ms inference and 50.15 ms total localization time, meeting real-time harvesting requirements.

**What are the implications of the main findings?**

- The model balances detection accuracy and speed under dense occlusion and variable illumination. Its real-time performance on a laptop demonstrates practical feasibility for integration into robotic harvesting systems.

**Abstract:**

To address the challenges of detecting sugar orange fruits in complex natural orchard environments—where fruits are frequently occluded by leaves and branches and may be mutually occluded due to dense growth, leading to missed detections, false positives, and low detection confidence—we propose an improved algorithm based on YOLOv8n, named YOLO-FSEP. A Spatial-Channel Synergistic Attention (SCSA) module is introduced into the main network to enhance feature extraction capabilities; the IoU loss function is replaced with Focal_SIOU to improve the detection accuracy for difficult samples; and an SE attention mechanism is embedded in the detection head, with the addition of a P6 high-resolution detection layer to optimize multi-scale object performance. Experimental results on a self-built sugar orange dataset show that, compared to the baseline YOLOv8n, the improved model achieves a 0.9% increase in accuracy, a 1.3% increase in recall, and a 3.2% increase in mAP50-95, while maintaining an inference speed of 62.6 FPS. To evaluate the model under dynamic conditions, we performed a 200-frame continuous test of the 3D localization pipeline on a laptop with a RealSense D435i camera. The average YOLO inference time was 49.90 ms, post-processing (depth extraction and 3D coordinate conversion) took 0.24 ms, and the total processing time was 50.15 ms. Given that the typical response time for a robotic arm’s single positioning operation is 100–200 ms, this real-time performance meets the dynamic localization requirements of sugar orange harvesting.

## 1. Introduction

China is the world’s largest producer of citrus fruits. As one of the major varieties, the sugar orange accounts for a large proportion of both the total citrus cultivation area and production. Guangxi is the country’s largest sugar orange-producing region [1,2]. Currently, sugar orange harvesting is still primarily done by hand. During the picking process, workers must repeatedly bend over, raise their arms, and tilt their heads while using shears; prolonged work of this nature can easily lead to occupational diseases such as lumbar muscle strain. During the peak ripening period, orchards must complete large-scale harvesting within a short timeframe, and the contradiction between rural labor shortages and low harvesting efficiency is becoming increasingly prominent. Therefore, applying object detection technology to the automated harvesting of sugar oranges holds significant research significance and practical value [3,4]. Citrus fruit target recognition technology integrates techniques such as visual image acquisition, image segmentation, and object detection. Its core lies in accurately identifying fruit targets within natural environments and providing three-dimensional coordinate data for automated harvesting by analyzing their spatial information [5]. Currently, citrus fruit recognition methods are primarily categorized into three types: traditional object recognition methods, machine learning methods, and deep learning methods based on convolutional neural networks (CNNs) [6,7]. In recent years, with the advancement of deep learning, CNN-based two-stage and single-stage algorithms have been widely applied in the field of fruit recognition and localization [8]. Representative two-stage algorithms include R-CNN [9], Fast R-CNN [10], and Faster R-CNN [11], among others. Representative single-stage algorithms include SSD [12] and the YOLO series, among others. Li et al. [13] proposed a chili pepper detection method that incorporates an attention mechanism into the YOLOv4-tiny model, combined with an adaptive spatial feature pyramid based on multi-scale prediction. This method achieved an AP score of 95.11%. Liu et al. [14] proposed an improved YOLOv5 model for the identification and counting of citrus fruits in orchards, achieving an AP score of 98.40%. Wang Yihan [15] proposed the LT-YOLOv7 model, which uses lightweight feature extraction network RepVGG as the backbone, introduces deep separable convolutions in the neck network, employs the channel attention mechanism ECA, and applies the softDIoU_NMS algorithm to optimize the selection of prediction boxes. Kong [16] and others incorporated the BiFormer module—a dynamic sparsity-aware attention mechanism—into the backbone network of YOLOv8s, added a small-object detection layer to its neck, and replaced the original loss function with an EIoU loss function. The improved YOLOv8s achieved an average accuracy of 94.7% for citrus fruit recognition. For pear detection, Fan Xiangpeng [17] built upon YOLOv11 and introduced a C2PSA module to enhance feature discrimination between fruits and leaves, achieving good recall and mAP50 in a pear orchard. For tomato detection, Tong Zhimin [18] proposed a lightweight YOLO-ELS algorithm based on YOLOv8n, which enhances edge features via EIEM, expands the receptive field with LSKA, improves occluded region response with SEAM, and accelerates convergence with Inner-GIoU loss, achieving a detection speed of 28.2 FPS on an edge platform. Although these methods have improved fruit detection performance to some extent, the fruit varieties involved (pear, tomato) differ significantly from sugar oranges in terms of morphology, growth density, and occlusion severity. Sugar oranges grow densely, with frequent mutual occlusion and lush foliage [19]; existing models still have shortcomings in detection accuracy and robustness. Therefore, this paper improves and optimizes the sugar orange detection method based on YOLOv8n, proposing the YOLO-FSEP algorithm to enable more effective sugar orange recognition and detection in complex orchard environments.

## 2. Materials and Methods

### 2.1. Construction of the Image Dataset

**Figure 1:**
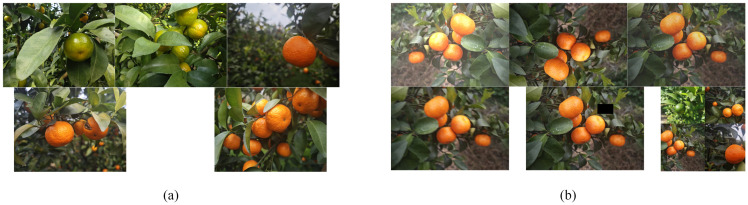
Samples from the sugar orange dataset and data augmentation results (**a**) Examples of some dataset samples. (**b**) Results of data-augmented samples.

This study selected sugar oranges as the research subject. Sugar orange images were collected on multiple occasions between September 2024 and January 2025 in sugar orange orchards around Guilin City, Guangxi Zhuang Autonomous Region. Images were captured using a Xiaomi 13 Pro smartphone (Xiaomi, Beijing, China) at a resolution of 4096 × 3072 pixels. To simulate real-world harvesting, we adopted a diverse collection strategy covering varying illumination (frontlight, sidelight, backlight), occlusion types (fruit, leaf, branch), fruit densities (single, multiple, dense), shooting heights (0.2–1 m), and fruit conditions (ripe, unripe, damaged). This diverse dataset helps enhance the model’s adaptability to complex environments. After manually screening and removing blurry and invalid images without fruit, the fruit was categorized into five classes: ripe sugar orange, unripe sugar orange, unripe sugar orange with occlusion, ripe sugar orange with occlusion, and rotten fruit. A portion of the samples is shown in Figure 1a. Annotation was performed using the Labelme annotation tool (v5.0.1). The processed dataset contains 2039 images of sugar oranges. The dataset was randomly split in an 8:1:1 ratio, with 1631 images used as the training set, 205 as the test set, and 203 as the validation set. Table 1 shows the detailed instance counts per category.

To improve model generalization and simulate complex orchard environments, the following online data augmentation strategies were applied during training: random brightness and contrast adjustment to simulate natural illumination changes and camera exposure uncertainty; random flipping to make the model focus on rotation-invariant features of the fruits; Gaussian noise addition to simulate sensor interference and act as a regularizer; motion blur to simulate image degradation caused by relative motion between the camera and target; random erasing to overlay black rectangular regions mimicking occlusion by leaves or branches; and mosaic mixing, which scales, crops, and stitches four images into a single training sample to effectively balance positive and negative sample ratios. All augmentations were generated dynamically and randomly in each training epoch without requiring offline storage of additional images. Figure 1b shows examples of the augmented samples.

### 2.2. YOLOv8n Model

The YOLOv8n algorithm is a single-stage object detection model released by Ultralytics in early 2023. The model architecture primarily consists of four components: the input layer, the main backbone network, the neck network, and the detection head. The backbone network comprises standard convolutions, C2f modules, downsampling, and spatial pyramids and is responsible for extracting multi-level features from the image, ranging from low-level edges to high-level semantic information. The neck network, comprising FPN, PAN, and operation modules (Upsample, Concat, C2f), is responsible for fusing features at different scales to enhance the detection capabilities for both small and large objects. The detection head employs a design without predefined anchor boxes [20], which is responsible for converting the fused feature maps into the final detection results.

### 2.3. Improvements to the YOLOv8n Model

**Figure 2:**
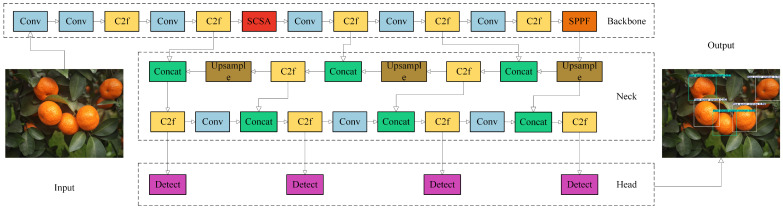
YOLO-FSEP network architecture.

To improve the model’s object detection accuracy in complex orchard environments, this paper introduces improvements to the YOLOv8n model. First, the backbone network is optimized by incorporating the Spatial Channel Synergistic Attention (SCSA) mechanism to enhance the model’s ability to focus on key regions and improve the extraction of sugar orange features. Second, the CIOU loss function is replaced with the Focal_SIOU loss function to improve the model’s handling of challenging samples, thereby enhancing the detection accuracy of occluded sugar oranges in complex backgrounds. Finally, we incorporate the P6 high-resolution detection head and the SE attention mechanism [21] into the detection head, further improving the model’s performance in handling large objects and complex backgrounds. The improved network model is named YOLO-FSEP, and its architecture is shown in Figure 2. This improvement fully considers various factors and enhances the model’s object detection capabilities in complex orchard environments.

#### 2.3.1. Spatial and Channel Co-Attention Module (SCSA)

In real-world target detection scenarios within orchards, complex environmental conditions such as image blurring, backlighting, and low light are common. In these situations, the model’s ability to extract spatial information from images is critical. The input feature maps first undergo spatial decoupling via the SMSA module, which utilizes deep shared 1D convolutions to extract spatial features of the sugar orange images at different scales. This step helps the model focus on the sugar orange fruit regions within the image and extract effective spatial information from the background. Additionally, to reduce semantic interference between different sub-features, the SMSA employs a group normalization strategy. This strategy is more suitable for the complex environments of sugar orange detection in this paper than batch normalization, effectively mitigating the impact of batch noise during model training. Finally, a spatial attention map is generated using the Sigmoid activation function. By assigning higher weights to key fruit regions, the model is better able to focus on the key features of the sugar oranges and suppress its attention to background areas such as leaves and branches.

In sugar orange object detection, features in the channel dimension often contain rich semantic information, such as the texture, color, and shape of the fruit. Since most fruits tend to occlude one another, using traditional channel attention mechanisms to extract global information via global average pooling can overlook important local features in the feature maps, leading to a loss of semantic information. This often prevents the model from effectively distinguishing features between different fruits; therefore, we address this issue by adopting a progressive channel self-attention mechanism. PCSA first performs layer-by-layer convolution and downsampling on the feature maps to preserve the primary semantic information of channel features while reducing redundant features. During the compression stage, PCSA incorporates spatial prior information passed from the SMSA module. Subsequently, it calculates the similarity relationships between channels using a single-head self-attention mechanism (SHSA). The compressed features are mapped to Query, Key, and Value. It captures semantic dependencies across different channels via a similarity matrix and generates a weighted output, thereby adaptively adjusting the response intensity of channels across the global scope. Subsequently, PCSA performs a nonlinear transformation and channel recalibration on the attention output. Through two layers of fully connected networks with Sigmoid activation, it generates a channel weight map and applies adaptive weighting to each channel based on these weights, achieving dynamic optimization and redistribution of features.

**Figure 3:**
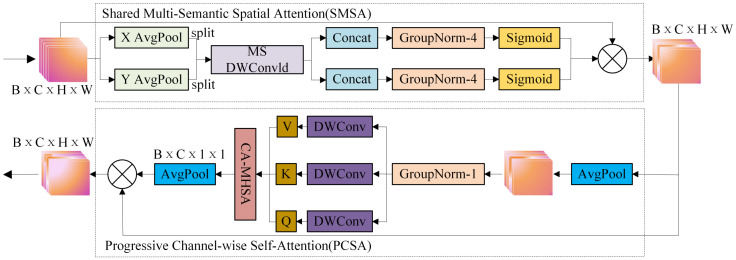
Structure of the SCSA module.

Through the combined action of SMSA and PCSA, the SCSA module achieves enhancement in both spatial and channel features. Specifically, SMSA is responsible for extracting salient regional features in the spatial dimension, effectively suppressing background interference and focusing on the key features of the sugar orange, while PCSA is responsible for semantic recalibration in the channel dimension, reinforcing the expression of global dependencies and local differences among features. The combination of these two approaches enhances the model’s ability to make accurate judgments under complex conditions such as fruit occlusion by leaves, varying light intensities, and fruit overlap, thereby reducing false negatives and false positives. This comprehensively improves the accuracy and robustness of the sugar orange object detection model. The SCSA module is illustrated in Figure 3.

#### 2.3.2. Focal_SIOU Loss Function

The CIOU loss function built into YOLOv8n cannot effectively address class imbalance and differences in detection difficulty among samples. In actual orchard detection environments, a significant portion of the sugar orange fruits are obscured by leaves or other fruits, which leads to a decline in detection performance. Therefore, this paper replaces YOLOv8n’s CIOU loss function with Focal_SIOU. Focal Loss was proposed to address the issue of class imbalance [22]. Its core idea is to use dynamic weighting to reduce the focus on easily classifiable samples (large objects and the background) and increase the focus on difficult samples (small targets and occluded objects). SIoU is an improved version of IoU that not only considers the overlapping area between predicted and ground-truth bounding boxes but also accounts for the distance between center points, differences in aspect ratios, and the shape of the bounding boxes. Traditional IoU loss yields a value of zero when boxes do not overlap, which prevents it from effectively capturing the spatial relationships between bounding boxes and thus affects gradient updates during model training. Therefore, the introduction of the Focal_SIOU loss function—which combines Focal Loss with SIoU—can simultaneously address the class imbalance issue in the sugar orange dataset and improve the localization accuracy of sugar oranges in complex backgrounds. The formula is shown as follows:

(1) $$Focal\_SIOU={\left(1-IoU\right)}^{\gamma }\cdot SIoU$$

where *IoU* represents the intersection over union between the predicted and ground-truth boxes, *SIoU* represents the spatial intersection over union incorporating center distance and aspect ratio, and *γ* is a tunable parameter controlling the model’s focus on hard samples.

The introduction of the *Focal_SIOU* loss function effectively improves the model’s detection accuracy, training speed, and robustness, enabling the model to better perform the sugar orange recognition and detection task.

#### 2.3.3. Improvements to the Detection Head

**Figure 4:**
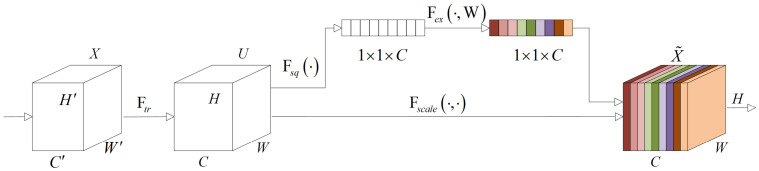
Schematic diagram of the SE module.

To further improve the model’s feature selection capability and detection accuracy without significantly increasing the number of model parameters, the SE attention mechanism is introduced in the detection head. First, after convolution operations, the SE module adaptively adjusts the weights of each channel to enhance feature channels related to the sugar orange class while suppressing background channels, allowing the detection head to focus on target information and improve detection accuracy. By enhancing feature channels related to the target class while reducing the weights of irrelevant and background channels, this approach increases accuracy and reduces false positives. Inserting the SE module into the detection head avoids the impact of premature weighting on feature extraction and reduces the computational burden on the backbone network. The structure of the SE module is shown in Figure 4.

**Figure 5:**
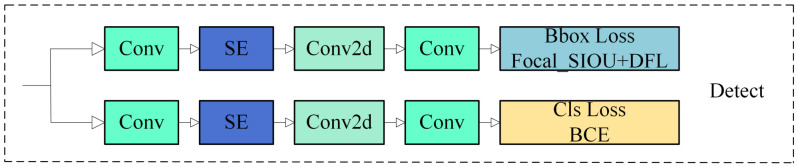
Improved detection head architecture incorporating SE attention and the P6 detection layer.

Analysis of the bounding box sizes in the dataset reveals that it contains targets of varying scales, including some large, occluded targets. In YOLO, the P5 layer is used to process larger targets; however, due to its low resolution, it may fail to effectively capture the details of large targets. When the target size far exceeds the receptive field of the feature map, inaccurate boundaries and information loss may occur. Therefore, adding a higher-resolution P6 detection head to process targets on higher-resolution feature maps can preserve more information. For occluded sugar orange models, the P6 detection head can better reconstruct the target’s shape and boundaries, reducing false negatives and false positives. Given that sugar oranges in orchards vary in size and that target dimensions fluctuate significantly depending on the shooting angle and position, incorporating the P6 detection head expands the coverage of the feature pyramid to enhance the model’s adaptability to large targets. The improved detection head is shown in Figure 5.

The four modules target different failure modes of sugar orange detection: SCSA addresses occlusion and low-light missed detections, Focal_SIOU improves localization of heavily occluded fruits, SE suppresses background false positives, and P6 recovers large mis-segmented fruits. Their synergistic design is tailored specifically to the dense occlusion and complex orchard conditions.

### 2.4. Experimental Environment

The experimental operating system was Windows 10, with an RTX 4090D (24 GB) GPU. The training environment consisted of CUDA 11.3 and Python 3.8. Model training parameters were as follows: initial learning rate of 0.01, final learning rate of 0.01, SGD optimizer enabled, optimizer weight decay of 0.0005, batch size of 32, and a total of 300 training epochs.

### 2.5. Evaluation Metrics

Recall (R), precision (P), mean average precision (mAP), and frames per second (FPS) were used as evaluation metrics. Precision indicates the model’s accuracy in detecting sugar oranges, as shown in Equation (2). Recall indicates the model’s completeness in detecting sugar oranges, as shown in Equation (3).

(2) $$P=\frac{TP}{TP+FP}$$

(3) $$R=\frac{TP}{TP+FN}$$

where *TP* represents the number of correctly detected sugar oranges, *FP* represents the number of false positives (background or incorrectly identified fruits), and *FN* represents the number of missed sugar oranges.

In the sugar orange harvesting task, object detection must balance both recognition accuracy and real-time efficiency. mAP is the core accuracy metric; a high mAP ensures that fruits are accurately identified and reliable harvesting coordinates are output, effectively improving the harvesting success rate and preventing erroneous robotic arm operations caused by misidentification. To minimize damage to fruit trees and fruits caused by erroneous robotic arm movements, the recognition accuracy P should be assigned a higher weight than the recall rate R in system evaluation. At the same time, FPS determines the model’s practicality in real-time scenarios; a high frame rate ensures the system’s timely response to environmental changes and the robotic arm’s rapid execution. Therefore, in the actual model selection process, priority is given to the mAP metric, followed by a comprehensive balancing of P, R, and FPS to achieve a balance between accuracy, speed, and resource consumption.

## 3. Results and Analysis

### 3.1. Performance Comparison of Mainstream Models

To select a baseline detection model suitable for the sugar orange harvesting task, we compared the performance of several mainstream detectors on our self-built dataset, including Faster R-CNN, SSD, YOLOv6, YOLOv8n, YOLOv10, YOLOv11, and YOLOv12. All models were trained and tested under identical experimental conditions, and the results are presented in Table 2. As shown in the table, YOLOv6 achieves the highest detection speed (85.4 FPS) but suffers from relatively low precision (66.0%) and mAP50-95 (64.0%), revealing a clear trade-off between speed and accuracy. YOLOv10 attains a moderate precision of 69.1%, yet its recall (69.3%) and mAP50-95 (62.5%) are both inferior to those of YOLOv8n. Faster R-CNN and SSD exhibit the lowest performance in terms of precision and mAP50-95 (66.5% and 55.6%, respectively), and their model sizes are also much larger (28.36 M and 24.15 M) with substantially higher computational costs (175 G and 120 G), indicating that traditional two-stage or anchor-based single-stage detection architectures struggle to achieve satisfactory results on the sugar orange dataset while being inefficient in terms of parameters and FLOPs. Notably, the more recent YOLOv11 and YOLOv12 models, despite their stronger performance on general benchmarks such as COCO, achieve mAP50-95 values of only 66.2% and 65.1%, respectively—both lower than the 68.5% of YOLOv8n—and their inference speeds are also considerably slower (60.6 and 49.5 FPS). Although YOLOv11 and YOLOv12 have slightly fewer parameters (2.58 M and 2.55 M) and lower GFLOPs (6.3 and 6.3) compared to YOLOv8n (3.00 M, 8.1 G), their degraded detection accuracy and slower inference on our dataset make them less suitable for the harvesting task. This performance degradation is likely attributable to the specific characteristics of sugar orange images, namely small fruit sizes, dense occlusion, and large illumination variations. The architectural design of YOLOv8n, including its C2f module and anchor-free detection head, appears to generalize better to such agricultural scenarios than the modifications introduced in later YOLO versions. In contrast, YOLOv8n demonstrates clear advantages in key accuracy metrics, achieving a precision of 73.4% and an mAP50-95 of 68.5%—both higher than any other compared model—while maintaining a competitive inference speed of 73.3 FPS with a compact model size (3.00 M, 8.1 G). Given the need to balance detection accuracy and real-time performance in harvesting tasks (where reliable fruit localization is critical for successful picking), we selected YOLOv8n as the baseline detection model for this study. Its superior accuracy, reasonable model complexity, and robust generalization on our dataset provide a solid foundation for subsequent model improvements.

### 3.2. Comparison of Different Loss Functions

To balance the weights among different samples, accelerate model convergence, and improve training efficiency and the algorithm’s generalization ability, it is crucial to select an appropriate loss function. In this paper, we replaced the CIOU loss function of YOLOv8n with DIOU, GIOU, SIOU, Focal_CIOU, Focal_DIOU, Focal_EIOU, Focal_GIOU, Focal_SIOU, and WIOU. The performance of different loss functions was analyzed using the same dataset and training parameters. Table 3 compares the results of the different loss functions. As shown in the table, compared to CIOU, Focal_SIOU increases R by 1.8%, mAP by 1.7%, and FPS by 1.486, while P decreases. Compared with other loss functions, this further demonstrates that introducing Focal_SIOU can enhance the model’s adaptability to occluded objects and complex backgrounds.

### 3.3. Module Ablation Test

To verify the impact of the improved modules on the model, we conducted comparative tests using our own dataset, with YOLOv8n as the baseline, on models incorporating different combinations of modules. The experimental results are shown in Table 4. As shown by the YOLO-S model in the table, the model’s mAP improved by 1.8%, indicating that the introduction of the SCSA module enhances the feature extraction capability of the backbone network and improves the representation of sugar orange fruit features. However, due to background suppression, the response to some edge-located fruits was weakened, resulting in a 3.2% decrease in R. As shown by the YOLO-E model, introducing the SE mechanism into the detection head module did not significantly increase the number of parameters but enhanced the model’s ability to focus on effective features. This reduced the computation of redundant features, thereby improving the model’s inference efficiency, ultimately boosting the inference speed to 86.2 FPS. As demonstrated by the YOLO-P model, the addition of the P6 high-resolution detection head enables the model to perform excellently in large-object detection tasks, with a 4.7% increase in P and a 1.7% increase in mAP, validating the effectiveness of the P6 architecture in enhancing the capture of details for large objects and overlapping fruits. As demonstrated by the YOLO-FS model, since the Focal_SIOU activation function suppresses easy samples and amplifies the gradients of difficult samples, and by combining it with SCSA to further amplify the channel responses of difficult samples (such as fine fruit stems and occluded edges), R is improved by 5.5%. However, when spatial suppression is too strong and the loss function is biased toward difficult examples, non-target structures near the boundaries may also be “over-signified,” leading to a decrease in P. As demonstrated by the YOLO-FEP model, combining P6 with Focal_SIOU significantly increases the model’s sensitivity to “suspicious regions” and substantially improves R. However, the higher resolution leads to more granular “background texture false positives.” When Focal weighting and SE are insufficient to fully suppress these, P decreases, resulting in an imbalance characterized by extremely high R and excessively low P. As observed in the YOLO-FSP model, the combination of SCSA and P6 simultaneously enhances “spatial saliency and high-resolution details,” making the model highly sensitive to challenging instances under Focal weighting. However, the lack of channel filtering (SE) in the detection heads makes it difficult to suppress redundant channels and pseudo-salient regions, resulting in compromised mAP and speed.

Although YOLO-FSEP introduces additional parameters and computational cost compared to YOLOv8n, the increase is modest: from 3.00 M to 5.05 M (+68%) and from 8.1 GFLOPs to 9.5 GFLOPs (+17%). In return, mAP50-95 improves by 3.2 percentage points. The inference speed on GPU remains 62.6 FPS. Therefore, the accuracy-complexity trade-off is acceptable.

Therefore, the YOLO-FSEP model is the optimal choice for sugar orange recognition and harvesting. During the training phase, Focal_SIOU redistributes the weights of easy and difficult samples, causing the model to focus more on occluded and hard-to-recognize sugar oranges, significantly improving recall and localization accuracy. The SCSA module enhances key regions of the fruit in the spatial dimension while suppressing redundant information in the channel dimension, thereby improving the effectiveness of feature extraction. The SE mechanism in the detection head module adaptively adjusts weights to enhance the representation of key features and reduce background interference, while simultaneously optimizing computational efficiency. The P6 detection head expands the scale range of the feature pyramid, improving the model’s recognition performance when dealing with large targets and complex environments. Through the synergistic interaction of these four modules, the YOLO-FSEP model achieves a 0.9% increase in P, a 1.3% increase in R, and a 3.2% increase in mAP50-95 compared to the baseline YOLOv8n model.

### 3.4. Visualization of Attention Heatmaps

**Figure 6:**
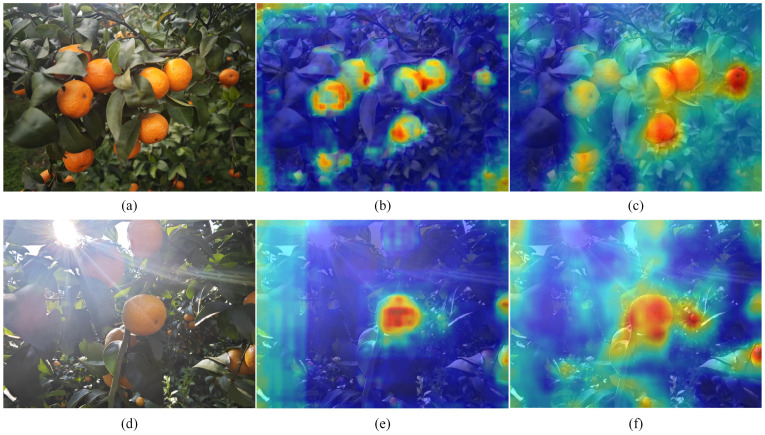
Comparison of heatmaps between YOLOv8n and YOLO-FSEP in different scenarios. (**a**) Original image of a dense occlusion scenario; (**b**) YOLOv8n heatmap; (**c**) YOLO-FSEP heatmap; (**d**) original image of a backlit scenario; (**e**) YOLOv8n heatmap; (**f**) YOLO-FSEP heatmap.

To visually compare the attention differences between the improved and original models, Grad-CAM++ was used to generate heatmaps, highlighting the image regions the model focuses on during specific predictions. Warm colors indicate high-attention areas, while cool colors indicate low-attention areas. The results are shown in Figure 6. Figure 6a–c show a densely occluded scene: the YOLOv8n heatmap (Figure 6b) fails to respond effectively to some targets, exhibiting attention loss, whereas the YOLO-FSEP heatmap (Figure 6c) provides more comprehensive attention coverage, focusing on the fruit area and effectively suppressing false attention to leaves and the background. Figure 6d–f show backlit scenes: the YOLOv8n heatmap (Figure 6e) responds strongly only to the fruit in the center of the image, demonstrating insufficient adaptability to backlighting; the YOLO-FSEP heatmap (Figure 6f) covers a larger area, with multiple fruits clearly highlighted. The above comparison demonstrates that the improvements in this paper endow the model with stronger semantic understanding and feature representation capabilities, as well as greater robustness to varying lighting conditions.

### 3.5. Comparative Analysis of Actual Detection Performance

**Figure 7:**
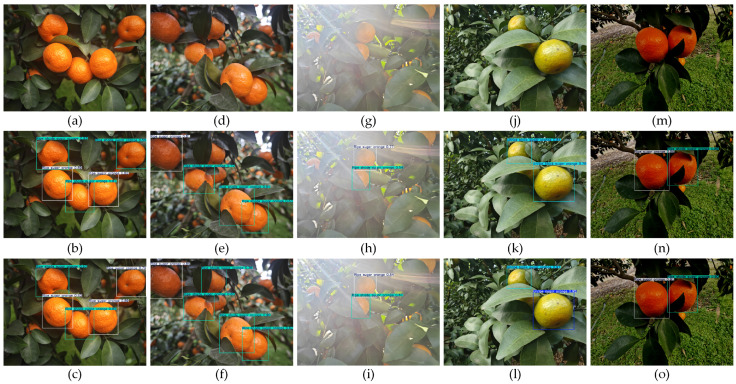
Comparison of detection performance between the YOLO-FSEP model and the YOLOv8n model. (**a**) Original image (sparse occlusion); (**d**) original image (dense occlusion); (**g**) original image (backlit); (**j**) original image (similar fruit-leaf color); (**m**) original image (low-light environment); (**b**,**e**,**h**,**k**,**n**) YOLOv8n detection results; (**c**,**f**,**i**,**l**,**o**) YOLO-FSEP detection results.

We selected images with occlusions and complex backgrounds from the dataset to conduct comparative tests between the YOLOv8n baseline model and the YOLO-FSEP improved model, using a confidence threshold of 0.5 and an IoU threshold of 0.45. The detection results are shown in Figure 7. In occlusion scenarios, YOLOv8n misclassified ripe sugar oranges as ripe sugar oranges with occlusion and failed to detect fruits with severe occlusion, whereas YOLO-FSEP, due to the introduction of the SCSA attention mechanism and the Focal_SIOU loss function, can more accurately focus on the fruit region and classify it correctly, while still reliably detecting fruits with severe occlusion. In backlit scenarios, both models can identify targets, but the detection confidence output by YOLO-FSEP is significantly higher than that of YOLOv8n. This demonstrates that the improved model in this paper can effectively reduce false negatives and false positives while maintaining high detection confidence when facing complex orchard environments such as dense fruit clusters, occlusion, and backlighting. This indicates that YOLO-FSEP possesses stronger target representation capabilities, adaptability to occlusion scenarios, and resistance to lighting variations, making it more reliable and stable for fruit detection tasks.

### 3.6. Analysis of Model Deployment Feasibility

**Figure 8:**
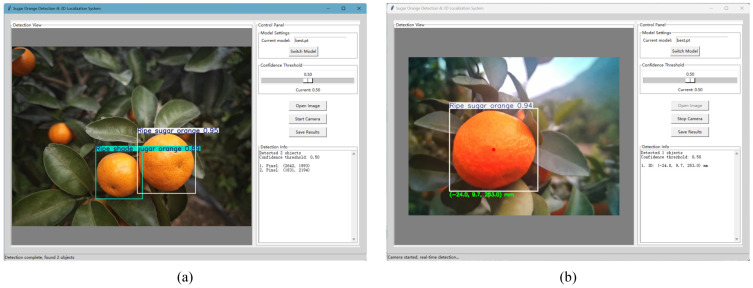
Interface of the sugar orange Recognition and 3D Localization System. (**a**) Image input detection mode. (**b**) Real-time detection mode with a depth camera.

To verify the engineering deployment capability of the YOLO-FSEP model in an actual harvesting system, the trained model was deployed on a laptop (Intel Core i7-8750H, 32 GB RAM, NVIDIA GTX 1060 graphics card). For the visual perception component, an Intel RealSense D435i depth camera (Intel, Santa Clara, CA, USA) was used to capture real-time color and depth video streams at a resolution of 640 × 480, as shown in Figure 8. To evaluate performance under continuous dynamic conditions, a 200-frame continuous test was conducted simulating the visual perception pipeline. The results show that the average YOLO-FSEP inference time is 49.90 ms, post-processing (including depth map center extraction, median filtering, and 3D coordinate conversion) takes 0.24 ms, and the total processing time from image input to fruit coordinate output is 50.15 ms. Considering that the allowable response time for a single positioning operation of a robotic arm’s end-effector is typically 100–200 ms, this real-time performance fully meets the dynamic positioning requirements of sugar orange harvesting operations. In summary, the YOLO-FSEP model can be effectively deployed in a practical harvesting robot vision system, providing reliable visual perception support for subsequent field harvesting.

## 4. Discussion

The proposed YOLO-FSEP model achieved effective progress in the sugar orange detection task. By integrating the SCSA attention, Focal_SIOU loss, SE attention, and P6 detection head, the model attained 71.7% mAP50-95 on the self-built dataset, a 3.2 percentage point improvement over YOLOv8n, while maintaining a GPU inference speed of 62.6 FPS and achieving an end-to-end 3D localization time of 50.15 ms on a laptop, meeting the real-time requirements of harvesting robots.

In recent years, significant progress has been made in YOLO-based fruit detection studies. For citrus detection, YOLO-MGP achieved 95.7% mAP50 by introducing the C2f-GLU module and coordinate attention [23]. For apple detection, an occlusion-aware lightweight model combined with SE attention reached 0.885 mAP50 with only 1.98 M parameters [19]. For peach detection, SDA-YOLO improved the localization accuracy of overlapping fruits using SPPF-LSKA and MPDIoU [24]. For tomato detection, FDA-YOLO achieved 83.4% mAP50 through multi-scale feature enhancement [25]. For passion fruit detection, G-YOLO-NK replaced the backbone network with GhostNet and incorporated knowledge distillation, achieving an average precision of 96.00% while keeping the model size at only 7.14 MB [26]. It should be noted that most of the above studies reported mAP50, whereas this work adopts the more stringent mAP50-95. According to common evaluation standards such as COCO, mAP50-95 is typically 20–30% lower than mAP50. Therefore, our 71.7% mAP50-95 is approximately equivalent to 85–90% mAP50, which is on par with the advanced methods mentioned above. Compared with these studies, the main characteristics of our work are: sugar oranges are smaller, grow in dense clusters causing more severe occlusion, and the color of unripe sugar oranges is highly similar to that of leaves, making the detection task significantly more challenging. Moreover, we adopt the stricter mAP50-95 metric and provide the actual end-to-end 3D localization latency on a laptop (50.15 ms), which is closer to the edge-computing scenario of agricultural harvesting. The combination of SCSA with a P6 detection head is applied to sugar orange detection in this work.

Despite the above progress, YOLO-FSEP still has certain limitations. Fully supervised learning requires a large amount of labeled data and has limited generalization ability to new orchards or different varieties. Future work will explore domain adaptation [27,28] or few-shot learning. The current dataset was collected only from the Guilin area, and cross-region generalization needs further validation. Extreme backlighting and highly similar fruit-leaf colors still lead to a small number of missed detections, which could be mitigated by illumination normalization or the introduction of instance segmentation.

## 5. Conclusions

This paper presented YOLO-FSEP, an improved YOLOv8n-based detection algorithm for sugar oranges in complex orchard environments. The model integrated SCSA attention, Focal_SIOU loss, SE attention, and a P6 detection head, achieving 71.7% mAP50-95 (3.2% improvement) at 62.6 FPS on a self-constructed dataset. Real-time performance was verified through a continuous test on a laptop, meeting the dynamic localization requirements for harvesting. The results demonstrate that YOLO-FSEP effectively balances detection accuracy and speed and is feasible for integration into practical sugar orange picking systems. Future work will focus on handling more severe occlusions and conducting field tests on integrated mobile robotic platforms.

## Figures and Tables

**Table 1:** Detailed instance counts per category in the sugar orange dataset.

Category	Images	Instances
Ripe	812	2147
Unripe	603	1689
Ripe occluded	342	876
Unripe occluded	198	502
Rotten	84	132
Total	2039	5346

**Table 2:** Comparison of different models.

Model	P/%	R/%	mAP50-95/%	FPS	Params/M	GFLOPs
Faster R-CNN	62.9	66.3	66.5	69.9	28.36	175
SSD	50.6	52.6	55.6	71.3	24.15	120
YOLOv6	66.0	77.2	64.0	85.4	4.23	11.8
YOLOv10	69.1	69.3	62.5	69.6	2.26	6.5
YOLOv8n	73.4	76.5	68.5	73.3	3.00	8.1
YOLOv11	71.5	74.2	66.2	60.6	2.58	6.3
YOLOv12	69.8	72.5	65.1	49.5	2.55	6.3

**Table 3:** Training results of different loss functions on the sugar orange dataset.

Loss Function	P/%	R/%	mAP50-95/%	FPS
CIOU	73.4	76.5	68.5	73.3
DIOU	70.5	72.8	67.8	75.6
EIOU	69.9	78.3	67.1	71.5
GIOU	62.1	82.6	69.3	81.2
SIOU	67.0	77.8	67.4	66.3
Focal_CIOU	65.2	80.0	69.0	72.6
Focal_DIOU	69.0	79.4	68.2	72.4
Focal_EIOU	63.8	83.6	67.1	74.4
Focal_GIOU	71.0	77.5	71.5	71.6
Focal_SIOU	71.3	78.3	70.2	74.8
WIOU	64.8	80.0	66.9	68.4

**Table 4:** Ablation experiments.

Model	Focal_SIOU	SCSA	Head_se	P6	P/%	R/%	mAP50-95/%	FPS	Params/M	GFLOPs
YOLOv8n					73.4	76.5	68.5	73.3	3.00	8.1
YOLO-F	✓				71.3	78.3	70.2	74.8	3.00	8.1
YOLO-S		✓			71.5	73.3	70.3	66.5	3.00	8.1
YOLO-E			✓		70.4	74.8	66.4	86.2	3.00	8.1
YOLO-P				✓	78.1	75.2	70.2	63.0	4.41	9.5
YOLO-FS	✓	✓			64.5	82.0	66.9	76.3	3.00	8.1
YOLO-FP	✓			✓	71.7	73.9	68.7	61.8	4.86	9.5
YOLO-FSE	✓	✓	✓		69.1	82.5	67.0	72.1	3.01	8.1
YOLO-FEP	✓		✓	✓	61.4	84.9	70.9	65.9	4.86	9.5
YOLO-FSP	✓	✓		✓	71.6	78.8	66.1	62.8	5.04	9.5
YOLO-FSEP	✓	✓	✓	✓	74.3	77.8	71.7	62.6	5.05	9.5

## Data Availability

The data presented in this study are available on request from the corresponding author.
